# Divergence from, and Convergence to, Uniformity of Probability Density Quantiles

**DOI:** 10.3390/e20050317

**Published:** 2018-04-25

**Authors:** Robert G. Staudte, Aihua Xia

**Affiliations:** 1 Department of Mathematics and Statistics, La Trobe University, Bundoora, VIC 3086, Australia; 2 School of Mathematics and Statistics, University of Melbourne, Parkville, VIC 3010, Australia

**Keywords:** convergence in *L_r_* norm, fixed point theorem, Kullback–Leibler divergence, relative entropy, semi-metric, uniformity testing, Primary 62E10, Secondary 62F03

## Abstract

We demonstrate that questions of convergence and divergence regarding shapes of distributions can be carried out in a location- and scale-free environment. This environment is the class of probability density quantiles (pdQs), obtained by normalizing the composition of the density with the associated quantile function. It has earlier been shown that the pdQ is representative of a location-scale family and carries essential information regarding shape and tail behavior of the family. The class of pdQs are densities of continuous distributions with common domain, the unit interval, facilitating metric and semi-metric comparisons. The Kullback–Leibler divergences from uniformity of these pdQs are mapped to illustrate their relative positions with respect to uniformity. To gain more insight into the information that is conserved under the pdQ mapping, we repeatedly apply the pdQ mapping and find that further applications of it are quite generally entropy increasing so convergence to the uniform distribution is investigated. New fixed point theorems are established with elementary probabilistic arguments and illustrated by examples.

## 1. Introduction

For each continuous location-scale family of distributions with square-integrable density, there is a probability density quantile (pdQ), which is an absolutely continuous distribution on the unit interval. Members of the class of such pdQs differ only in *shape*, and the asymmetry of their shapes can be partially ordered by their Hellinger distances or Kullback–Leibler divergences from the class of symmetric distributions on this interval. In addition, the tail behaviour of the original family can be described in terms of the boundary derivatives of its pdQ. Empirical estimators of the pdQs enable one to carry out inference, such as robust fitting of shape parameter families to data; details are in [1].

The Kullback–Leibler directed divergences and symmetrized divergence (KLD) of a pdQ with respect to the uniform distribution on [0,1] is investigated in Section 2, with remarkably simple numerical results, and a map of these divergences for some standard location-scale families is constructed. The ‘shapeless’ uniform distribution is the center of the pdQ universe, as is explained in Section 3, where it is found to be a fixed point. A natural question of interest is to find the invariant information of the pdQ mapping, that is, the conserved information after the pdQ mapping is applied. To this end, it is necessary to repeatedly apply the pdQ mapping to extract the information. Numerical studies indicate that further applications of the pdQ transformation are generally entropy increasing, so we investigate the convergence to uniformity of repeated applications of the pdQ transformation, by means of fixed point theorems for a semi-metric. As the pdQ mapping is not a contraction, the proofs of the fixed point theorems are through elementary probabilistic arguments rather than the classical contraction mapping principle. Our approach may shed light on future research in the fixed point theory. Further ideas are discussed in Section 4.

## 2. Divergences between Probability Density Quantiles

### 2.1. Definitions

Let F denote the class of cumulative distribution functions (cdfs) on the real line R and for each F∈F define the associated *quantile function* of *F* by Q(u)=inf{x:F(x)≥u}, for 0<u<1. When the random variable *X* has cdf *F*, we write X∼F. When the density function $f=F^{\prime }$ exists, we also write X∼f. We only discuss *F* as absolutely continuous with respect to Lebesgue measure, but the results can be extended to the discrete and mixture cases using suitable dominating measures.

**Definition** **1.** *Let $F{\;}^{\prime }=\left\{F\in F\;:\;f=F^{\prime }\;\mathrm{exists}\;\mathrm{and}\;\mathrm{is}\;\mathrm{positive}\right\}$. For each $F\in F{\;}^{\prime }$, we follow [2] and define the* quantile density *function $q\left(u\right)=Q^{\prime }\left(u\right)=1/f\left(Q\left(u\right)\right)$. Parzen called its reciprocal function fQ(u)=f(Q(u)) the* density quantile *function. For $F\in F{\;}^{\prime }$, and U uniformly distributed on [0,1], assume $\kappa =E\left[fQ\left(U\right)\right]=\int f^{2}\left(x\right)\;dx$ is finite; that is, f is square integrable. Then, we can define the* continuous pdQ of F by $f^{*}\left(u\right)=fQ\left(u\right)/\kappa$, 0<u<1. Let $F{\;}^{\prime *}\subset F{\;}^{\prime }$ denote the class of all such F.

Not all *f* are square-integrable, and this requirement for the mapping $f\to f^{*}$ means that $F{\;}^{\prime *}$ is a proper subset of $F{\;}^{\prime }.$ The advantages of working with $f^{*}$s over *f*s are that they are free of location and scale parameters; they ignore flat spots in *F* and have a common bounded support. Moreover, $f^{*}$ often has a simpler formula than *f*; see Table 1 for examples.

**Remark** **1.** Given that a pdQ $f^{*}$ exists for a distribution with density f, then so does the cdf $F^{*}$ and quantile function $Q^{*}={\left(F^{*}\right)}^{-1}$ associated with $f^{*}$. Thus, a monotone transformation from X∼F to $X^{*}∼F^{*}$ exists; it is simply $X^{*}=m\left(X\right)=Q^{*}\left(F\left(X\right)\right)$. For the Power(b) distribution of Table 1, $f_{b}^{*}=f_{b^{*}}$, where $b^{*}=2-1/b$, so $m_{b}\left(x\right)=Q_{b}^{*}\left(F_{b}\left(x\right)\right)=x^{b/b^{*}}=x^{b^{2}/\left(2b-1\right)}.$ For the normal distribution with parameters μ,σ, it is $m_{\mu ,\sigma }\left(x\right)=\Phi \left(\left(x-\mu \right)/\sqrt{2}\;\sigma \right).$ In general, an explicit expression for $Q^{*}$ that depends only on f or F (plus location-scale parameters) need not exist.

### 2.2. Divergence Map

Next, we evaluate and plot the [3] divergences from uniformity. The [3] divergence of density $f_{1}$ from density $f_{2}$, when both have domain [0,1], is defined as

$$I\left(f_{1}:f_{2}\right):=\int_{0}^{1}\ln\left(f_{1}\left(u\right)/f_{2}\left(u\right)\right)\;f_{1}\left(u\right)\;du=E\left[\ln\left(f_{1}\left(U\right)/f_{2}\left(U\right)\right)\;f_{1}\left(U\right)\right],$$

where *U* denotes a random variable with the uniform distribution U on [0,1]. The divergences from uniformity are easily computed through

$$I\left(U:f^{*}\right)=-\int_{0}^{1}\ln\left(f^{*}\left(u\right)\right)\;du=-E\left[\ln\left(f^{*}\left(U\right)\right)\right]$$

and

$$I\left(f^{*}:U\right)=\int_{0}^{1}\ln\left(f^{*}\left(u\right)\right)\;f^{*}\left(u\right)\;du=E\left[\ln\left(f^{*}\left(U\right)\right)\;f^{*}\left(U\right)\right].$$

Kullback ([4], p. 6) interprets $I(f^{*}:U)$ as the mean evidence in one observation $V∼f^{*}$ for $f^{*}$ over U; it is also known as the *relative entropy* of $f^{*}$ with respect to U. The terminology directed divergence for $I(f_{1}:f_{2})$ is also sometimes used ([4], p. 7) with ‘directed’ explained in ([4], pp. 82, 85); see also [5] in this regard.

Table 1 shows the quantile functions of some standard distributions, along with their pdQs, associated divergences $I\left(U:f^{*}\right),I\left(f^{*}:U\right)$ and symmetrized divergence (KLD) defined by $J\left(U,f^{*}\right):=I\left(U:f^{*}\right)+I\left(f^{*}:U\right)$. The last measure was earlier introduced in a different form by [6].

**Definition** **2.** Given pdQs $f_{1}^{*}$, $f_{2}^{*}$, let $d\left(f_{1}^{*},f_{2}^{*}\right):=\sqrt{I\left(f_{1}^{*}:f_{2}^{*}\right)+I\left(f_{2}^{*}:f_{1}^{*}\right)}$. Then, d is a semi-metric on the space of pdQs; i.e., d satisfies all requirements of a metric except the triangle inequality. Introducing the coordinates $\left(s_{1},s_{2}\right)=\left(\sqrt{I(U:f^{*})}\;,\sqrt{I(f^{*}:U)}\right)$, we can define the distance from uniformity of any $f^{*}$ by the Euclidean distance of $\left(s_{1},s_{2}\right)$ from the origin (0,0), namely $d(U,f^{*})$.

**Figure 1:**
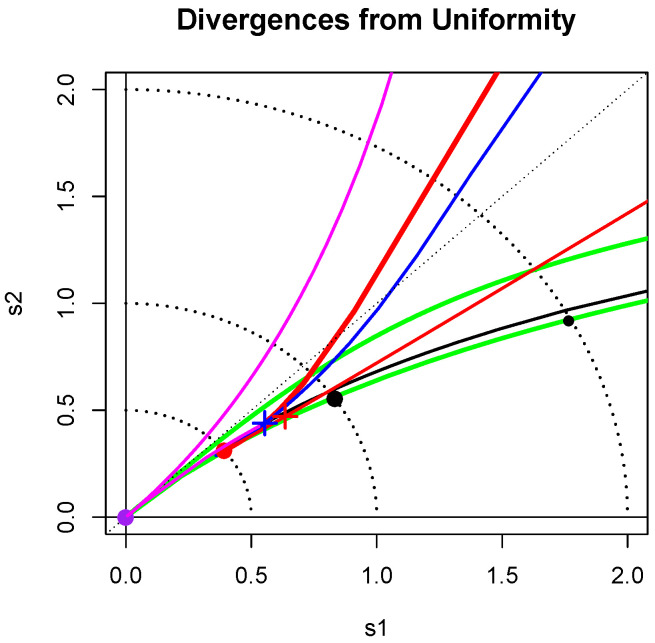
Divergence from uniformity. The loci of points $\left(s_{1},s_{2}\right)=\left(\sqrt{I(U:f^{*})}\;,\sqrt{I(f^{*}:U)}\;\right)$ is shown for various standard families. The large disks correspond respectively to the symmetric families: uniform (purple), normal (red) and Cauchy (black). The crosses correspond to the asymmetric distributions: exponential (blue) and standard lognormal (red). More details are given in Section 2.2.

**Remark** **2.** This d does not satisfy the triangle inequality: for example, if U,N and C denote the uniform, normal and Cauchy pdQs, then d(U,N)=0.5,d(N,C)=0.4681 but d(U,C)=1; see Table 1 and Figure 1. However, d can provide an informative measure of distance from uniformity.

Figure 1 shows the loci of points $\left(s_{1},s_{2}\right)$ for some continuous shape families. The light dotted arcs with radii 1/2, 1 and 2 are a guide to these distances from uniformity. The large discs in purple, red and black correspond to U, N and C. The blue cross at distance $1/\sqrt{2}$ from the origin corresponds to the exponential distribution. Nearby is the standard lognormal point marked by a red cross. The lower red curve is nearly straight and is the locus of points corresponding to the lognormal shape family.

The chi-squared(ν), ν>1, family also appears as a red curve; it passes through the blue cross when ν=2, as expected, and heads toward the normal disc as ν→∞. The Gamma family has the same locus of points as the chi-squared family. The curve for the Weibull(β) family, for 0.5<β<3, is shown in blue; it crosses the exponential blue cross when β=1. The Pareto(*a*) curve is shown in black. As *a* increases from 0, this line crosses the arcs distant 2 and 1 from the origin for $a=(2\sqrt{2}\;+1)/7\approx 0.547$ and $a=(\sqrt{5}\;-1)/2\approx 1.618$, respectively, and approaches the exponential blue cross as a→∞.

The Power(*b*) or Beta(b,1) for b>1/2 family is represented by the magenta curve of points moving toward the origin as *b* increases from 1/2 to 1, and then moving out towards the exponential blue cross as b→∞. For each choice of α>0.5, β>0.5 the locus of the Beta(α,β) pdQ divergences lies above the chi-squared red curve and mostly below the power(*b*) magenta curve; however, the U-shaped Beta distributions have loci above it.

The lower green line near the Pareto black curve gives the loci of root-divergences from uniformity of the Tukey(λ) with λ<1, while the upper green curve corresponds to λ≥1. It is known that the Tukey(λ) distributions, with λ<1/7, are good approximations to Student’s *t*-distributions for ν>0, provided λ is chosen properly. The same is true for their corresponding pdQs ([1], Section 3.2). For example, the pdQof $t_{\nu }$ with ν=0.24 degrees of freedom is well approximated by the choice λ=−4.063. Its location is marked by the small black disk in Figure 1; it is of distance 2 from uniformity. The generalized Tukey distributions of [7] with two shape parameters also fill a large funnel shaped region (not marked on the map) emanating from the origin and just including the region bounded by the green curves of the Tukey symmetric distributions.

### 2.3. Uniformity Testing

There are numerous tests for uniformity, but as [8] points out, many are undermined by the common practice of estimating location-scale parameters of the null and/or alternative distributions when in fact it is assumed that these distributions are known exactly. In practice, this means that if a test for uniformity is preceded by a probability integral transformation including parameter estimates, then the actual levels of such tests will not be those nominated unless (often complicated and model-specific) adjustments are made. Examples of such adjustments are in [9,10].

Given a random sample of *m* independent, identically distributed (i.i.d.) variables, each from a distribution with density *f*, it is feasible to carry out a nonparametric test of uniformity by estimating the pdQ with a kernel density estimator $\hat{f_{m}^{*}}$ and comparing it with the uniform density on [0,1] using any one of a number of metrics or semi-metrics. Consistent estimators $\hat{f_{m}^{*}}$ for $f^{*}$ based on normalized reciprocals of the quantile density estimators derived in [11] are available and described in (Staudte [1], Section 2). Note that such a test compares an *arbitrary* uniform distribution with an *arbitrary* member of the location-scale family generated by *f*; it is a test of shape only. Preliminary work suggests that such a test is feasible. However, an investigation into such omnibus nonparametric testing procedures, including comparison with bootstrap and other kernel density based techniques found in the literature, such as [12,13,14,15,16,17], is beyond the scope of this work.

## 3. Convergence of Density Shapes to Uniformity via Fixed Point Theorems

The transformation $f\to f^{*}$ of Definition 1 is quite powerful, removing location and scale and moving the distribution from the support of *f* to the unit interval. A natural question of interest is to find the information in a density that is invariant after the pdQ mapping is applied. To this end, it is necessary to repeatedly apply the pdQ mapping to extract the information. Examples suggest that another application of the transformation $f^{2*}:={\left(f^{*}\right)}^{*}$ leaves less information about *f* in $f^{2*}$ and hence it is closer to the uniform density. Furthermore, with *n* iterations $f^{(n+1)*}:={\left(f^{n*}\right)}^{*}$ for n≥2, it seems that no information can be conserved after repeated *-transformation so we would expect that $f^{n*}$ converges to the uniform density as n→∞. An R script [18] for finding repeated *-iterates of a given pdQ is available as Supplementary Material.

### 3.1. Conditions for Convergence to Uniformity

**Definition** **3.** *Given $f\in F{\;}^{\prime }$, we say that f is of* *-order *n* if $f^{*},f^{2*},\ldots ,f^{n*}$ exist but $f^{(n+1)*}$ does not. When the infinite sequence ${\left\{f^{n*}\right\}}_{n\geq 1}$ exists, it is said to be of infinite *-order.

For example, the Power(3/4) family is of *-order 2, while the Power(2) family is of infinite *-order. The $\chi_{\nu }^{2}$ distribution is of finite *-order for 1<ν<2 and infinite *-order for ν≥2. The normal distribution is of infinite *-order.

We write $\mu_{n}:=\int_{-\infty }^{\infty }{\left\{f\left(y\right)\right\}}^{n}\;dy$, $\kappa_{n}=\int_{0}^{1}{\left\{f^{n*}\left(x\right)\right\}}^{2}\;dx$, n≥1, and $\kappa_{0}=\int_{-\infty }^{\infty }{\left\{f\left(x\right)\right\}}^{2}\;dx$. The next proposition characterises the property of infinite *-order.

**Proposition** **1.** *For $f\in F{\;}^{\prime }$ and m≥1, the following statements are equivalent:*

*(i)*  $\mu_{m+2}<\infty$,
*(ii)*  $\mu_{j}<\infty$ for all 1≤j≤m+2,
*(iii)*  $\kappa_{j}<\infty$ and $\kappa_{j}=\frac{\mu_{j}\mu_{j+2}}{\mu_{j+1}^{2}}$ for all 1≤j≤m.

In particular, f is of infinite *-order if and only if $\mu_{n}<\infty$, n≥1.

**Proof** **of** **Proposition** **1.** For each i,n≥1, provided all terms below are finite, we have the following recursive formula

(1) $$\nu_{n,i}:=\int {\left\{f^{n*}\left(x\right)\right\}}^{i}\;dx=\frac{1}{\kappa_{n-1}^{i}}\;\nu_{n-1,i+1},$$

giving

(2) $$\kappa_{n}=\frac{1}{\prod_{j=0}^{n-1}\kappa_{j}^{n+1-j}}\;\mu_{n+2}.$$

(i) ⇒ (ii) For 1≤j≤m+2,

$$\begin{array}{cc} \mu_{j} & =\int_{-\infty }^{\infty }{\left\{f\left(x\right)\right\}}^{j}1_{\left\{f(x)>1\right\}}dx+\int_{-\infty }^{\infty }{\left\{f\left(x\right)\right\}}^{j}1_{\left\{f(x)\leq 1\right\}}dx\\ & \leq \int_{-\infty }^{\infty }{\left\{f\left(x\right)\right\}}^{m+2}dx+\int_{-\infty }^{\infty }f\left(x\right)dx=\mu_{m+2}+1<\infty . \end{array}$$

(ii) ⇒ (iii) Use (2) and proceed with induction for 1≤n≤m.

(iii) ⇒ (i) By Definition 1, $\kappa_{1}<\infty$ means that $\kappa_{0}<\infty$. Hence, (i) follows from (2) with n=m. ☐

Next, we investigate the involutionary nature of the *-transformation.

**Proposition** **2.** Let $f^{*}$ be a pdQ and assume $f^{2*}$ exists. Then, $f^{*}∼U$ if and only if $f^{2*}∼U$.

**Proof** **of** **Proposition** **2.** For r>0, we have

(3) $$\int_{0}^{1}|f^{2*}{\left(u\right)-1|}^{r}\;du=\frac{1}{\kappa_{1}^{r}}\int_{0}^{1}{\left|f^{*}\left(x\right)-\kappa_{1}\right|}^{r}f^{*}\left(x\right)\;dx.$$

If $f^{*}\left(u\right)∼U$, then $\kappa_{1}=1$ and (3) ensures $\int_{0}^{1}{\left|f^{2*}\left(u\right)-1\right|}^{r}du=0$, so $f^{2*}\left(u\right)∼U$.

Conversely, if $f^{2*}\left(u\right)∼U$, then using (3) again gives $\int_{0}^{1}{\left|f^{*}\left(x\right)-\kappa_{1}\right|}^{r}f^{*}\left(x\right)\;dx=0$. Since $f^{*}\left(x\right)>0$ a.s., we have $f^{*}\left(x\right)=\kappa_{1}$ a.s. and this can only happen when $\kappa_{1}=1$. Thus, $f^{*}∼U$, as required. ☐

Proposition 2 shows that the uniform distribution is a fixed point in the Banach space of integrable functions on [0,1] with the $L_{r}$ norm for any r>0. It remains to show that $f^{n*}$ has a limit and that the limit is the uniform distribution. It was hoped that the classical machinery for convergence in Banach spaces ([19], Chapter 10) would prove useful in this regard, but the *-mapping is not a contraction. For this reason, although there are many studies of fixed point theory in metric and semi-metric spaces (see, e.g., [20] and references therein), the fixed point Theorems 1, 2 and 3 shown below do not seem to be covered in these general studies. Moreover, our proofs are purely probabilistic and non-standard in this area. For simplicity, we use $\overset{L_{r}}{⟶}$ to stand for the convergence in $L_{r}$ norm and $\overset{P}{⟶}$ for convergence in probability as n→∞.

**Theorem** **1.** For $f\in F{\;}^{\prime }$ with infinite *-order, the following statements are equivalent:

*(i)*  $f^{n*}\overset{L_{2}}{⟶}1$;
*(ii)*  For all r>0, $f^{n*}\overset{L_{r}}{⟶}1$;
*(iii)*  $\frac{\mu_{n}\mu_{n+2}}{\mu_{n+1}^{2}}\to 1$ as n→∞.

**Remark** **3.** Notice that $\mu_{n}=E\left\{f^{*}{\left(U\right)}^{n-1}\right\}$, n≥1, are the moments of the random variable $f^{*}\left(U\right)$ with U∼U. Theorem 1 says that the convergence of $\left\{f^{n*}:\;n\geq 1\right\}$ is purely determined by the moments of $f^{*}\left(U\right)$. This is rather puzzling because it is well known that the moments do not uniquely determine the distribution ([21], p. 227), meaning that different distributions with the same moments have the same converging behaviour. However, if f is bounded, then $f^{*}\left(U\right)$ is a bounded random variable so its moments uniquely specify its distribution ([21], pp. 225–226), leading to stronger results in Theorem 2.

**Proof of** **Theorem 1** It is clear that (ii) implies (i).

(i) ⇒ (iii): By Proposition 1, $\kappa_{n}=\frac{\mu_{n}\mu_{n+2}}{\mu_{n+1}^{2}}$. Now,

(4) $$\int_{0}^{1}{\left\{f^{n*}\left(x\right)-1\right\}}^{2}\;dx=\kappa_{n}-1,$$

so (iii) follows immediately.

(iii) ⇒ (ii): It suffices to show that $f^{n*}\overset{L_{r}}{⟶}1$ for any integer r≥4. To this end, since for a,b≥0, ${\left|a-b\right|}^{r-2}\leq a^{r-2}+b^{r-2}$, we have from (4) that

(5) $$\int_{0}^{1}{\left|f^{n*}\left(x\right)-1\right|}^{r}dx\leq \int_{0}^{1}{\left(f^{n*}\left(x\right)-1\right)}^{2}\left(f^{n*}{\left(x\right)}^{r-2}+1\right)dx=\nu_{n,r}-2\nu_{n,r-1}+\nu_{n,r-2}+\kappa_{n}-1,$$

where, as before, $\nu_{n,r}=\int_{0}^{1}{\left\{f^{n*}\left(x\right)\right\}}^{r}dx$. However, applying (1) gives

$$\nu_{n,r}=\frac{\mu_{n+r}}{\kappa_{n-1}^{r}\kappa_{n-2}^{r+1}\ldots \kappa_{0}^{n+r-1}}$$

and (2) ensures

$$\mu_{n+r}=\kappa_{n+r-2}\kappa_{n+r-3}^{2}\ldots \kappa_{0}^{n+r-1},$$

which imply

$$\nu_{n,r}=\kappa_{n+r-2}\kappa_{n+r-3}^{2}\ldots \kappa_{n}^{r-1}\to 1$$

as n→∞. Hence, it follows from (5) that $\int_{0}^{1}{\left|f^{n*}\left(x\right)-1\right|}^{r}dx\to 0$ as n→∞, completing the proof. ☐

We write $∥g∥=\sup_{x}\left|g\left(x\right)\right|$ for each bounded function *g*.

**Theorem** **2.** *If f is bounded, then*

*(i)*  for all n≥0, $∥f^{(n+1)*}∥\leq ∥f^{n*}∥$ and the inequality becomes equality if and only if $f^{n*}∼U$;
*(ii)*  $f^{n*}\overset{L_{r}}{⟶}1$ for all r>0.

**Proof** **of** **Theorem** **2.** It follows from (4) that $\kappa_{n}\geq 1$ and the inequality becomes equality if and only if $f^{n*}∼U$.

(i) Let $Q^{n*}$ be the inverse of the cumulative distribution function of $f^{n*}$, then $f^{(n+1)*}\left(u\right)=\frac{f^{n*}\left(Q^{n*}\left(u\right)\right)}{\kappa_{n}}\leq \frac{∥f^{n*}∥}{\kappa_{n}}$, giving $∥f^{(n+1)*}∥\leq \frac{∥f^{n*}∥}{\kappa_{n}}\leq ∥f^{n*}∥$. If $f^{n*}∼U$, then Proposition 2 ensures that $f^{(n+1)*}∼U$, so $∥f^{(n+1)*}∥=∥f^{n*}∥$. Conversely, if $∥f^{(n+1)*}∥=∥f^{n*}∥$, then $\kappa_{n}=1$, so $f^{n*}∼U$.

(ii) It remains to show that $\kappa_{n}\to 1$ as n→∞. In fact, if $\kappa_{n}↛1$, since $\kappa_{n}\geq 1$, there exist a δ>0 and a subsequence $\left\{n_{k}\right\}$ such that $\kappa_{n_{k}}\geq 1+\delta$, which implies

(6) $$\frac{\mu_{n_{k}+2}}{\mu_{n_{k}+1}}=\prod_{i=0}^{n_{k}}\kappa_{i}\geq {\left(1+\delta \right)}^{k}\to \infty \;as\;k\to \infty .$$

However, $\frac{\mu_{n_{k}+2}}{\mu_{n_{k}+1}}\leq ∥f∥<\infty$, which contradicts (6). ☐

**Theorem** **3.** For $f\in F{\;}^{\prime }$ with infinite *-order such that $\left\{\mu_{n}\mu_{n+2}\mu_{n+1}^{-2}:\;n\geq 1\right\}$ is a bounded sequence, then the following statements are equivalent:

(i*)  $f^{n*}\overset{P}{⟶}1$;
*(ii)*  For all r>0, $f^{n*}\overset{L_{r}}{⟶}1$;
*(iii)*  $\mu_{n}\mu_{n+2}\mu_{n+1}^{-2}\to 1$ as n→∞.

**Proof** **of** **Theorem** **3.** It suffices to show that (i*) implies (iii). Recall that $\kappa_{n}=\mu_{n}\mu_{n+2}\mu_{n+1}^{-2}$. For each subsequence $\left\{\kappa_{n_{k}}\right\}$, there exists a converging sub-subsequence $\left\{\kappa_{n_{k_{i}}}\right\}$ such that $\kappa_{n_{k_{i}}}\to b$ as i→∞. It remains to show that b=1. To this end, for δ>1, we have

(7) $$\begin{array}{c} \int_{0}^{1}\left|f^{(n_{k_{i}}+1)*}\left(x\right)-1\right|1_{\left\{\left|f^{(n_{k_{i}}+1)*}\left(x\right)-1\right|\leq \delta \right\}}dx\\ =\frac{1}{\kappa_{n_{k_{i}}}}\int_{0}^{1}\left|f^{(n_{k_{i}})*}\left(x\right)-\kappa_{n_{k_{i}}}\right|f^{(n_{k_{i}})*}\left(x\right)1_{\left\{\left|f^{\left(n_{k_{i}}\right)*}\left(x\right)-\kappa_{n_{k_{i}}}\right|\leq \delta \kappa_{n_{k_{i}}}\right\}}dx. \end{array}$$

(i*) ensures that

$$\left|f^{(n_{k_{i}}+1)*}-1\right|\overset{P}{⟶}0,\;f^{(n_{k_{i}})*}\left|f^{(n_{k_{i}})*}-\kappa_{n_{k_{i}}}\right|\overset{P}{⟶}\left|1-b\right|,\;1_{\left\{\left|f^{(n_{k_{i}})*}\left(x\right)-\kappa_{n_{k_{i}}}\right|\leq \delta \kappa_{n_{k_{i}}}\right\}}\overset{P}{⟶}1$$

as i→∞, so applying the bounded convergence theorem to both sides of (7) to get 0=|1/b−1|, i.e., b=1. ☐

**Remark** **4.** We note that not all distributions are of infinite *-order so the fixed point theorems are only applicable to a proper subclass of all distributions.

### 3.2. Examples of Convergence to Uniformity

The main results in Section 3.1 cover all the standard distributions with infinite *-order in [22,23]. In fact, as observed in the Remark after Theorem 1 that the convergence to uniformity is purely determined by the moments of $f^{*}\left(U\right)$ with U∼U, we have failed to construct a density such that $\left\{f^{n*}:\;n\geq 1\right\}$ does not converge to the uniform distribution. Here, we give a few examples to show that the main results in Section 3.1 are indeed very convenient to use.

**Example** **1.** Power function family.

From Table 1, the Power(b) family has density $f_{b}\left(x\right)=bx^{b-1},\;0<x<1$, so it is of infinite *-order if and only if b≥1. As $f_{b}$ is bounded for b≥1, Theorem 2 ensures that $f_{b}^{n*}$ converges to the uniform in $L_{r}$ for any r>0.

**Example** **2.** Exponential distribution.

Suppose $f\left(x\right)=e^{x},\;\;x<0$. f is bounded, so Theorem 2 says that $f^{n*}$ converges to the uniform distribution as n→∞. By symmetry, the same result holds for $f\left(x\right)=e^{-x},\;\;x>0$.

**Example** **3.** Pareto distribution.

The Pareto(a) family, with a>0, has $f_{a}\left(x\right)=ax^{-a-1}$ for x>1, which is bounded, so an application of Theorem 2 yields that the sequence ${\left\{f_{a}^{n*}\right\}}_{n\geq 1}$ converges to the uniform distribution as n→∞.

**Example** **4.** Cauchy distribution.

The pdQ of the Cauchy density is given by $f^{*}\left(u\right)=2\sin^{2}\left(\pi u\right)$, 0<u<1, see Table 1; it retains the bell shape of f. It follows that $F^{*}\left(t\right)=t-\sin\left(2\pi t\right)/\left(2\pi \right),$ for 0<t<1. It seems impossible to obtain an analytical form of $f^{n*}$ for n≥2. However, as f is bounded, using Theorem 2, we can conclude that $f^{n*}$ converges to the uniform distribution as n→∞.

**Example** **5.** Skew-normal.

*A skew-normal distribution [17,24] has the density of the form*

f(x)=2ϕ(x)Φ(αx),x∈R,

where α∈R is a parameter, ϕ and *Φ*, as before, are the density and cdf of the standard normal distribution. When α=0, f is reduced to the standard normal so it is possible to obtain its $\left\{f^{n*}\right\}$ by induction and then derive directly that $f^{n*}$ converges to the uniform distribution as n→∞. However, the general form of skew-normal densities is a lot harder to handle and one can easily see that the density is bounded and so Theorem 2 can be employed to conclude that $f^{n*}$ converges to the uniform distribution as n→∞.

**Example** **6.** Let f(x)=−lnx, x∈(0,1). Then, $\mu_{n}=n!$ and $\kappa_{n}=\frac{n+2}{n+1}\to 1$ as n→∞, so we have from Theorem 1 that, for any r>0, $f^{n*}$ converges in $L_{r}$ norm to constant 1 as n→∞.

## 4. Discussion

The pdQ, transformation from a density function *f* to $f^{*}$ extracts the important information of *f* such as its asymmetry and tail behaviour and ignores the less critical information such as gaps, location and scale, and thus provides a powerful tool in studying the shapes of density functions. We found the directed divergences from uniformity of the pdQs of many standard location-scale families and used them to make a map locating each shape family relative to others and giving its distance from uniformity. It would be of interest to find the pdQs of other shape families, such as the skew-normal of Example 5; however, a simple expression for this pdQ appears unlikely given the complicated nature of its quantile function. Nevertheless, the [25] skew-normal family should be amenable in this regard because there are explicit formulae for both its density and quantile functions. To obtain the information conserved in the pdQ transformation, we repeatedly applied the transformation and found the limiting behaviour of repeated applications of the pdQ mapping. When the density function *f* is bounded, we showed that each application lowers its modal height and hence the resulting density function $f^{*}$ is closer to the uniform density than *f*. Furthermore, we established a necessary and sufficient condition for $f^{n*}$ converging in $L_{2}$ norm to the uniform density, giving a positive answer to a conjecture raised in [1]. In particular, if *f* is bounded, we proved that $f^{n*}$ converges in $L_{r}$ norm to the uniform density for any r>0. The fixed point theorems can be interpreted as follows. As we repeatedly apply the pdQ transformation, we keep losing information about the shape of the original *f* and will eventually exhaust the information, leaving nothing in the limit, as represented by the uniform density, which means no points carry more information than other points. Thus, the pdQ transformation plays a similar role to the difference operator in time series analysis where repeated applications of the difference operator to a time series with a polynomial component lead to a white noise with a constant power spectral density ([26], p. 19). We conjecture that every almost surely positive density *g* on [0,1] is a pdQ of a density function, hence uniquely representing a location-scale family. This is equivalent to saying that there exists a density function *f* such that $g=f^{*}$. When *g* satisfies $\int_{0}^{1}\frac{1}{g(t)}dt<\infty$, one can show that the cdf *F* of *f* can be uniquely (up to location-scale parameters) represented as $F\left(x\right)=H^{-1}\left(H\left(1\right)x\right)$, where $H\left(x\right)=\int_{0}^{x}\frac{1}{g(t)}dt$ (Professor A.D. Barbour, personal communication). The condition $\int_{0}^{1}\frac{1}{g(t)}dt<\infty$ is equivalent to saying that *f* has bounded support and it is certainly not necessary, e.g., g(x)=2x for x∈[0,1] and $f\left(x\right)=e^{x}$ for x<0 (see Example 2 in Section 3.2).

## 5. Conclusions

In summary, the study of shapes of probability densities is facilitated by composing them with their own quantile functions, which puts them on the same finite support where they are absolutely continuous with respect to Lebesgue measure, and thus amenable to metric and semi-metric comparisons. In addition, we showed that further applications of this transformation, which intuitively reduces information and increases the relative entropy, is generally valid but requires a non-standard approach for proof. Similar results are likely to be obtainable in the multivariate case. Further research could investigate the relationship between relative entropy and tail-weight or distance from the class of symmetric pdQs.

## Figures and Tables

**Table 1:** Quantiles of some distributions, their pdQs and divergences. In general, we denote $x_{u}=Q\left(u\right)=F^{-1}\left(u\right)$, but for the normal F=Φ with density ϕ, we use $z_{u}=\Phi^{-1}\left(u\right)$. The logistic quantile function is only defined for u≤0.5, but it is symmetric about u=0.5. Lognormal(σ) represents the lognormal distribution with shape parameter σ. The quantile function for the Pareto is for the Type II distribution with shape parameter *a*, and the pdQ is the same for Type I and Type II Pareto models.

	Q(u)	$\;f^{*}\left(u\right)$		$I(U:f^{*})$	$I(f^{*}:U)$	$J(U,f^{*})$
Normal	$z_{u}$	$2\sqrt{\pi }\;\phi \left(z_{u}\right)$		0.153	0.097	0.250
Logistic	ln(u/(1−u))	6u(1−u)		0.208	0.125	0.333
Laplace	ln(2u),u≤0.5	2min{u,1−u}		0.307	0.193	0.500
$t_{2}$	$\frac{2u-1}{{\left\{2u\left(1-u\right)\right\}}^{1/2}}$	$\frac{2^{7}{\left\{u\left(1-u\right)\right\}}^{3/2}}{3\pi }$		0.391	0.200	0.591
Cauchy	tan{π(u−0.5)}	$2\sin^{2}\left(\pi u\right)$		0.693	0.307	1.000
Exponential	−ln(1−u)	2(1−u)		0.307	0.193	0.500
Gumbel	−ln(−ln(u))	−4uln(u)		0.191	0.116	0.307
Lognormal (σ)	$e^{\;\sigma \;z_{u}}$	$\frac{2\sqrt{\pi }\;}{e^{\sigma^{2}/4}}\;\phi \left(z_{u}\right)\;e^{-\sigma \;z_{u}}$		$\frac{\sigma^{2}}{4}+\frac{1}{2}-\ln\left(\sqrt{2}\right)$	-	$\frac{1}{4}+\frac{3\sigma^{2}}{8}$
Pareto (*a*)	${\left(1-u\right)}^{-1/a}$	$\frac{2a+1}{a}\;{\left(1-u\right)}^{1+1/a}$		$\frac{1+a}{a}-\ln\left(2+\frac{1}{a}\right)$	-	$\frac{{\left(1+a\right)}^{2}}{a(1+2a)}$
Power (*b*)	$u^{1/b}$	$\frac{2b-1}{b}\;u^{1-1/b}$		$\frac{b-1}{b}-\ln\left(2-\frac{1}{b}\right)$	-	$\frac{{\left(b-1\right)}^{2}}{b(2b-1)}$

## Supplementary Materials

An R script entitled StaudteXiaSupp.R, which is available online at http://www.mdpi.com/1099-4300/20/5/317/s1, enables the reader to plot successive iterates of the pdQ transformation on any standard probability distribution available in R.
